# Latest Developments to Manufacture Metal Matrix Composites and Functionally Graded Materials through AM: A State-of-the-Art Review

**DOI:** 10.3390/ma16041746

**Published:** 2023-02-20

**Authors:** Marta Ostolaza, Jon Iñaki Arrizubieta, Aitzol Lamikiz, Soraya Plaza, Naiara Ortega

**Affiliations:** Department of Mechanical Engineering, University of the Basque Country (UPV/EHU), Plaza Ingeniero Torres Quevedo, 48013 Bilbao, Spain

**Keywords:** multi-material, additive manufacturing, laser directed energy deposition, metal matrix composites, functionally graded materials

## Abstract

Multi-material structure fabrication has the potential to address some critical challenges in today’s industrial paradigm. While conventional manufacturing processes cannot deliver multi-material structures in a single operation, additive manufacturing (AM) has come up as an appealing alternative. In particular, laser-directed energy deposition (L-DED) is preferred for multi-material AM. The most relevant applications envisioned for multi-material L-DED are alloy design, metal matrix composites (MMC), and functionally graded materials (FGM). Nonetheless, there are still some issues that need to be faced before multi-material L-DED is ready for industrial use. Driven by this need, in this literature review, the suitability of L-DED for multi-material component fabrication is first demonstrated. Then, the main defects associated with multi-material L-DED and current opportunities and challenges in the field are reported. In view of the industrial relevance of high-performance coatings as tools to mitigate wear, emphasis is placed on the development of MMCs and FGMs. The identified challenges include—but are not limited to—tightly controlling the composition of the multi-material powder mixture injected into the melt pool; understanding the influence of the thermal history of the process on microstructural aspects, including the interactions between constituents; and studying the in-service behaviours of MMCs and FGMs with regard to their durability and failure modes.

## 1. Introduction to Metal Additive Manufacturing

According to the International Standard ISO/ASTM 52900:2021, additive manufacturing (AM) is the “process of joining materials to make parts from 3D model data, usually layer upon layer, as opposed to subtractive manufacturing and formative manufacturing methodologies” [1]. Multiple materials can be processed through AM, namely polymers and resins [2], ceramics [3], and metals [4].

### 1.1. Industrial Context of Metal AM

In terms of the industrial relevance and market data, metal AM is experiencing significant growth, having reached turnover of 1.51 billion EUR in 2018 [5]. Moreover, according to SmarTech and General Electric, it is expected to reach 5.4 billion USD in 2027 [6]. The rapid development of the metal AM market has been mainly driven by the medical, dental, and aerospace industries, where the popularity of additive technologies is major, and additively built-up components are already being employed for end-use purposes. Even if AM has been widely adopted for prototyping applications, functional component manufacturing is gradually establishing itself in the industry too. Moreover, AM has been demonstrated to provide great benefits in repair applications [7], on-demand spare part manufacturing, or low-run and custom production.

In short, AM has emerged as a state-of-the-art technology and it is quickly gaining momentum in the industry, owing to the significant research efforts and advances made during the last decades. Nowadays, it is considered to be a reliable and efficient technology for manufacturing fully dense structural components. Additionally, it has become an industrially-viable technology on account of the lower cost of industrial lasers, the availability of high-performance computing software, and the high-quality feedstock technology. Most AM technologies, and particularly metal AM processes, have reached a critical acceptance level in the industry. Moreover, some of them have reached a fully certified production stage in terms of the technology readiness levels (TRL) [8]. Nowadays, AM technologies are completely immersed in many industries such as the manufacture of medical implants or high-performance components in the aerospace sector [9].

However, there is still a great need for AM, more specifically metal AM, to be included in additional industrial fields for high-end parts manufacturing, as it is many times stuck in the research phase. For instance, the capabilities of AM in the automotive and machine tool sectors have been widely demonstrated in many research papers [10,11,12,13]. Nevertheless, companies have not included such advances yet. Due to the disruptive character of metal AM processes, many manufacturers still identify their integration as a potential risk. Indeed, an adaptation of the current supply chain is required to fully benefit from AM’s potential for high-end part production. Lastly, the standardisation of processes is required rather than the certification of the individual components. Although AM is considered to be part of the Fourth Industrial Revolution [14], making it an essential part of industry 4.0 [15], the business strategies and certification policies have not kept pace with the growth of the technology [14]. Consequently, there is still a lot of work in progress in the area of standardisation, which will contribute to AM being integrated into the whole industry.

The main AM process categories currently employed for metal AM are powder bed fusion (PBF) and directed energy deposition (DED) processes [16]. Many others have the potential for metal manufacturing, e.g., sheet lamination (SL) or binder jetting (BJT). For instance, BJT is slowly gaining relevance in metal AM, and several potential applications are envisioned. Nonetheless, this technology is still far from obtaining fully dense parts. Therefore, it cannot be considered a valid technology for high-responsibility applications yet. In short, these technologies have lower industrial relevance. Consequently, they are not discussed in this review and only PBF and DED process categories are further described.

### 1.2. Metal AM Materials and Properties

The main characteristics and applications of typical metal AM materials are shown in Table 1. The available material range is continuously expanding due to the exhaustive research in metal AM, whilst multi-material AM is also being explored.

The main limitation of metal AM is the fact that fusion is involved in the building process; hence, non-weldable and non-castable materials are difficult to process using fusion AM methods [17]. However, much research is being carried out to face this challenge, and non-weldable materials have been successfully deposited [18,19,20]. AM strategies based on the precise control of the energy input and the tuning of the thermal history of the parts during build-up seem to be the key aspects for tackling this issue [21].

**Table 1:** Most industrially extended materials for AM [22,23,24].

Material	Main Characteristics	Application
Tool steels	− High toughness − High ductility − Good resistance to deformation	Tooling for cutting, forming, or shaping processes
Stainless steels	− Corrosion resistance − High ductility − High strength	Structural and corrosion-resistant applications
Titanium alloys	− High specific strength − Exceptional corrosion resistance − High fracture toughness − Excellent fatigue resistance − Good mechanical properties at high T − Low coefficient of thermal expansion − Good biocompatibility	Aerospace, automotive, naval and biomedical applications
Aluminium alloys	− Low density − High specific strength − High ductility and toughness − High thermal and electrical conductivities	Aerospace, automotive, construction and consumer goods
Nickel-based alloys	− Excellent tensile and creep strengths − Good mechanical properties at high T° − High-temperature oxidation resistance − High hardness and toughness − Low coefficient of thermal expansion − Good weldability and formability	Aerospace and jet engine, steam turbine, petrochemical, energy, and cryogenic applications
Cobalt-based alloys	− High hardness − Exceptional wear resistance − Good corrosion resistance − Good high-temperature performance	Aerospace and jet engines, petrochemical, oil and gas, medical implants, wear- resistant applications
Copper alloys	− High electrical conductivity − High thermal conductivity − Good mechanical properties	Fusion reactors, rocket engine, microelectronics

Additionally, metallurgical differences have been found between additively and conventionally manufactured components. The high residual stresses and lack of integrity are critical for high-performance applications, particularly for those that require good resistance to high-temperature fatigue [9]. This is crucial in aerospace applications, where injectors and other complex parts are now reaching the certification stage, but other critical components, e.g., turbine blades, are still in the early development phase [8].

Historically, single materials and well-defined commercial metal alloys have dominated the engineering market. However, the multi-material ability of AM processes has unlocked a new research direction concerning the build-up of multi-material structures [25]. In terms of metal and metal–ceramic multi-material component production, the presence of AM in the industrial market is still minor. In this regard, several challenges remain unsolved, which inhibit its widespread industrialisation [26]. Therefore, the occurrences in the industrial field of 3D-printed metal–ceramic multi-materials remain anecdotal. Moreover, most appearances of metal–ceramic additively manufactured parts have been in the context of mining and oil and gas industries.

### 1.3. Main Metal Additive Manufacturing Processes

In this section, the main metal AM categories, namely PBF and DED, are discussed. The fundamentals of the most popular metal AM processes are briefly explained, and a critical comparison is provided, with an emphasis on multi-material AM.

#### 1.3.1. Powder Bed Fusion

The powder bed fusion (PBF) term refers to the “*additive manufacturing process in which thermal energy selectively fuses regions of a powder bed*” [1]. This category comprises several technologies, which include but are not limited to laser PBF (L-PBF) and electron beam PBF (EB-PBF). However, they are all based on the same working principle.

**Figure 1:**
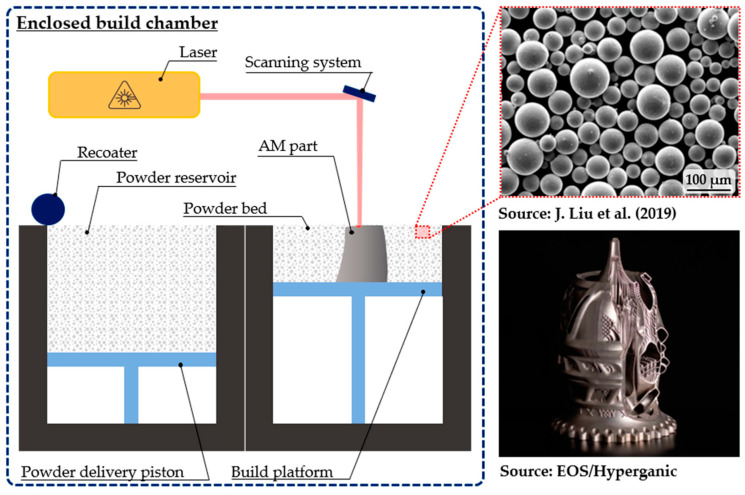
L-PBF system, feedstock, and example application [28,29].

PBF processes are currently widely accepted in the industry and well-established in the aerospace and medical fields. In particular, L-PBF (popularly referred to as selective laser melting or SLM) is probably the most extended metal AM process. In Figure 1, a typical L-PBF system is schematically illustrated. On the left-hand side, the main system components are shown. On the right–hand side, the typical feedstock required for this process and an example of its application are also included.

**Figure 2:**
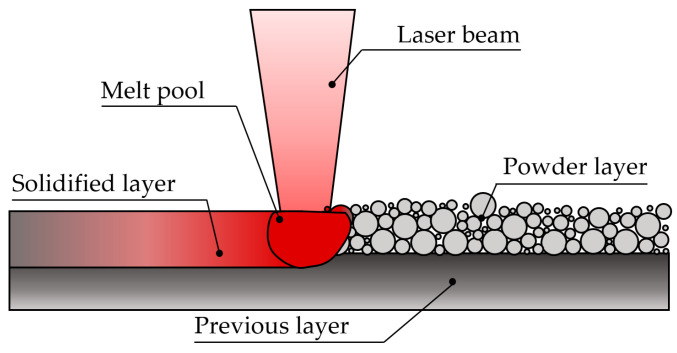
Working principle of the L-PBF process.

The L-PBF process can be described as follows:

1. The powder delivery piston pushes the powder reservoir up and the recoater spreads a layer of fresh powder onto either the building platform (first layer) or the previously deposited layers (next layers) to form the powder bed. This powder bed should be properly distributed to ensure the densification of the manufactured parts;
2. The laser delivery system irradiates a laser beam, which is guided by the scanning system, along the path predefined by the sliced 3D model data. As shown in Figure 1, the feedstock powder is quite fine and the typical diameter range for the powder is 10–60 µm [27,28]. During this process, a melt pool is generated, whose depth needs to exceed the layer thickness to guarantee proper bonding of the layers (Figure 2);
3. Once the layer is finished and the pattern is solidified, the build platform goes down and steps 1 and 2 are repeated. By overlapping subsequent layers (typically 30- to 90-µm-thick) and iteratively repeating this cycle, the AM part is formed, achieving results similar to the aerospike nozzle shown in Figure 1 [29]. Note that the whole process is carried out in an enclosed build chamber with an inert gas atmosphere to avoid the oxidation and cross-contamination of the parts.

#### 1.3.2. Directed Energy Deposition

Directed energy deposition (DED) processes are those “additive manufacturing processes in which focused thermal energy is used to fuse materials by melting as they are being deposited” [1]. In these processes, the feedstock is typically in the form of a powder or wire, and the thermal source is a laser or an electric arc, although an electron beam can be employed too. Three processes are traditionally included in this category: laser-directed energy deposition (L-DED), in which powder or wire is used as feedstock and a laser as a heat source; electron beam free-form fabrication (EBF3), in which wire is used as the feedstock and an electron beam as the heat source; and wire and arc additive manufacturing (WAAM), in which a plasma arc is used as the energy source and wire as the feedstock. Owing to the higher popularity of L-DED and WAAM, they are further described in this section.

**Figure 3:**
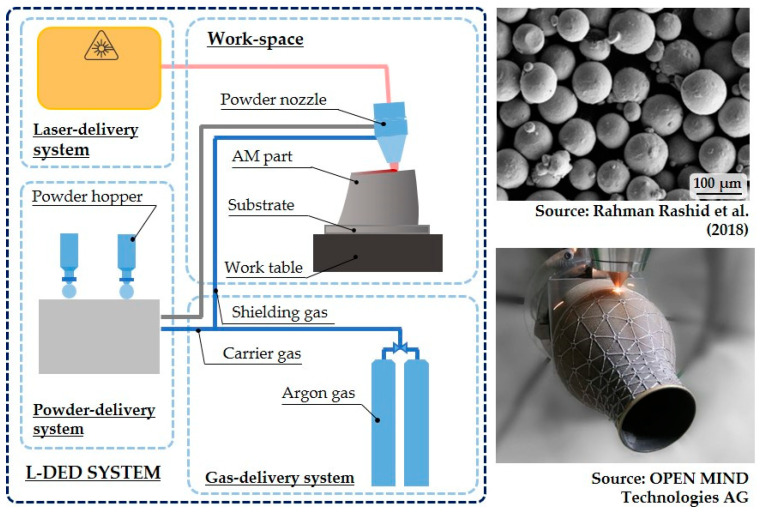
L-DED system, feedstock, and example application [34,35].

Laser-directed energy deposition (L-DED) is the most extended DED process in the industry. The typical set-up of an L-DED system is illustrated in Figure 3.

**Figure 4:**
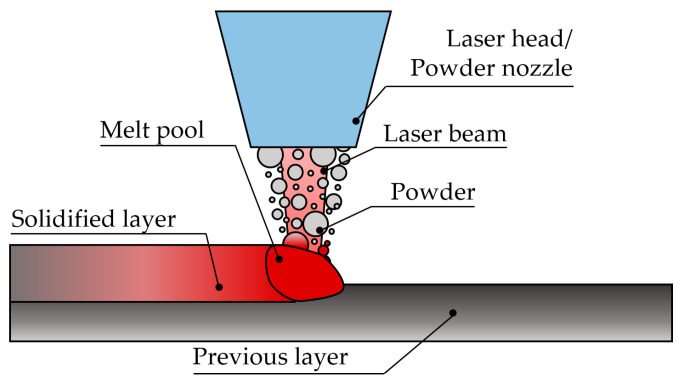
L-DED working principle.

Most of the time, the feedstock material is provided in powder form, and it is fully melted and well-densified in order to produce high-quality components [30]. In terms of the multi-material ability, one of the main advantages of powder L-DED is the wider material availability as compared to wire feedstock. Another advantage of powder L-DED is the possibility for in situ alloy design, as various elemental powders can be fed to produce the desired alloy. Due to the ease of dynamically modifying the resulting composition of the powder mixture during the build-up, powder L-DED is the preferred solution for multi-material AM research. Indeed, wire L-DED does not share such flexibilities [31]. Consequently, in this review, the focus is placed on powder L-DED. The powder L-DED is described as follows:

1. A laser beam is focused onto a substrate where a melt pool is created;
2. Simultaneously, powder particles are injected into the melt pool and the material is progressively added to the substrate (Figure 4). The powder diameter range is typically 40–150 µm [32,33], as shown in Figure 3 [34];
3. The powder particles are supplied by the powder feeder and dragged by an inert gas to the nozzle. Additionally, a shielding gas is supplied by the nozzle, typically argon, to create a local protective atmosphere, where the fusion and solidification process takes place. In this manner, oxidation of the added material is avoided, or at least minimised;
4. There is a relative movement between the laser head or powder nozzle and the substrate, thereby depositing a thin layer corresponding to the cross-section of the desired geometry;
5. After a layer is completed, the deposition of the following layer starts, hence building a three-dimensional component layer-by-layer. In this manner, not only can whole components be built, but also additional features can be added to a preform, as is the case of the part shown in Figure 3 [35].

Considering the growing interest of the industry and researchers in multi-material structures, the greatest advantage of the L-DED process seems to be its peerless multi-material ability and its capability for in situ modification of the feedstock composition. Additional nozzles or hoppers can be introduced into the feeding system to feed several alloys simultaneously [36]. In this manner, a gradual switch from one material to another is possible and the manufacturing of functionally graded materials (FGM) is unlocked. In addition, considering that the feedstock is in the form of powder, the in situ alloy design can also be achieved by directly mixing the elemental constituents [37]. Lastly, being a point-by-point manufacturing process, the material microstructure and composition can be tailored and specific location-dependent physical properties can be attained. For instance, graded deposition has been employed to combine dissimilar materials by matching the coefficients of thermal expansion (CTEs) between them. Additionally, the build-up of components aiming for novel mechanical properties through new alloy designs or composite materials has been demonstrated [38].

Wire arc additive manufacturing (WAAM) is nowadays gaining popularity in both industry and research owing to its capability to deliver defect-free and structurally sound large components, which cannot be manufactured through other metal AM processes. WAAM has been investigated since the 1990s [39]. However, at that time, it did not receive as much attention as compared to the rest of the metal AM processes [40].

**Figure 5:**
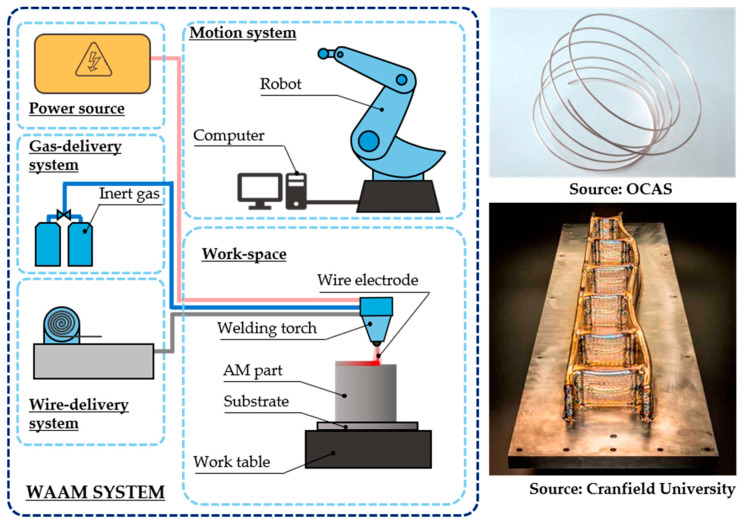
WAAM system, feedstock, and example application [41,42].

WAAM is, as the name implies, a wire-based metal AM process, in which an electric arc acts as the heat source [39]. Therefore, traditional arc welding processes are the basis of WAAM, and gas metal arc welding (GMAW), plasma arc welding (PAW), or gas tungsten arc welding (GTAW) are commonly employed. Nevertheless, GMAW is the preferred choice for WAAM. In Figure 5, a scheme of a WAAM system is shown. However, alternative configurations are also possible, as WAAM is an open architecture process. Examples of a wire used for WAAM [41] and a titanium wing spar manufactured using WAAM for BAE systems [42] are also shown in Figure 5.

**Figure 6:**
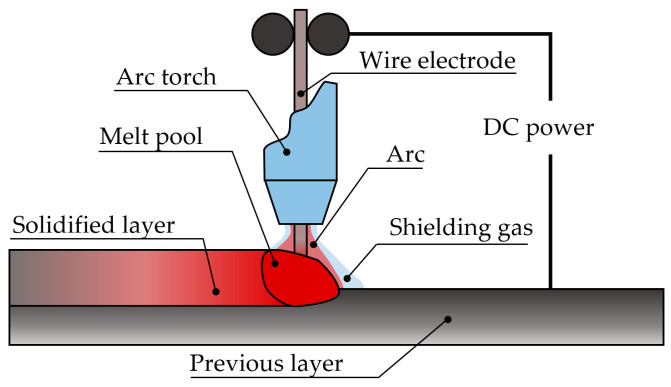
WAAM working principle.

The GMAW-based WAAM process is described as follows [43]:

1. An electric arc is struck between the substrate and the consumable wire, leading to the formation of a melt pool, as shown in Figure 6. This fusion process occurs under the protection of a shielding gas, typically argon or helium [44];
2. Then, as the wire is pushed into the melt pool, the material is fused and solidified. The consumable wire is continuously supplied. Simultaneously, the robotic arm moves the welding head and a clad is formed;
3. By properly overlapping clads and by overlaying subsequent layers, the AM component is generated.

#### 1.3.3. Critical Comparison of the Main Metal AM Processes

**Figure 7:**
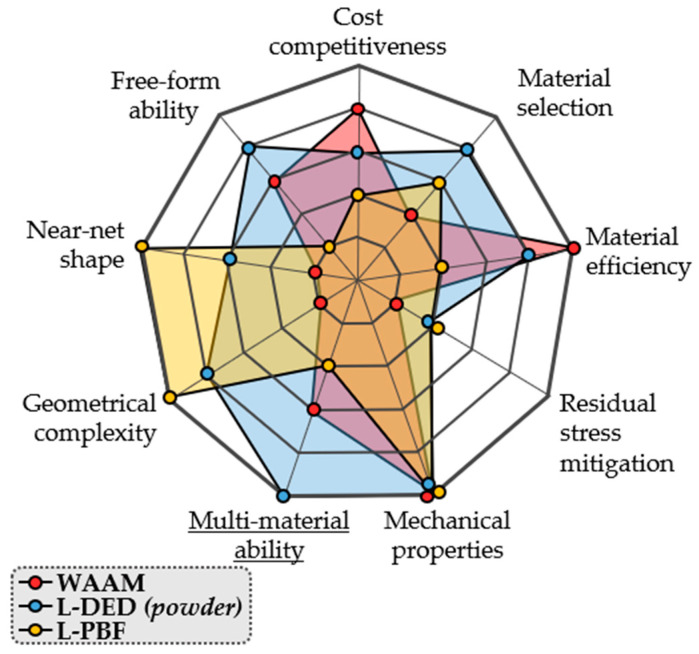
Qualitative comparison between L-PBF, L-DED, and WAAM. Adapted from [45].

To conclude, qualitative and quantitative comparisons of the above processes are shown in Figure 7 and Table 2, respectively.

From a qualitative perspective, nine different aspects are considered, namely the economical aspect (“cost competitiveness”), the material processability or material range that they can be applied to (“material selection”), the efficiency in terms of the material consumption (“material efficiency”), the ability (or lack thereof) to mitigate residual stresses (“residual stress mitigation”), the quality in terms of the mechanical properties of the AM components (“mechanical properties”), the geometric flexibility of the process (“geometrical complexity”), the amount of post-processing needed after the AM process (“near-net-shape”), the capability for material addition in different geometrical surfaces (“free-form ability”), and the straight-forward ability to produce multi-material structures (“multi-material ability”).

In Table 2, a summarised comparison between the three main metal AM processes is presented. This comparison relies on key indicators of the quality and applicability of metal AM processes. Namely, the maximum part dimension, the typical surface finish and dimensional accuracy attainable, the productivity in terms of the build rate, and the densification of each process are assessed.

Based on Figure 7 and Table 2, it is concluded that while good mechanical properties and fully dense components can be achieved using all processes, each process has its specific field of application. Firstly, the employment of L-PBF has an inherent part dimension restriction. Indeed, it is limited by the volume of the enclosed chamber where the building process takes place, while L-DED and WAAM have no such limitation. Additionally, WAAM permits much higher deposition rates, meaning better efficiency can be achieved in terms of productivity. Nonetheless, as far as the part complexity is concerned, WAAM is only suited for medium-low complexity geometries, while both L-PBF and L-DED have the capability for manufacturing more complex components. However, the L-PBF process is better suited for high-precision applications, where a good surface finish, dimensional tolerances, and reduced post-processing are required.

Another relevant aspect that differentiates metal AM processes is whether they have a straight-forward multi-material ability or not. While potentially all L-PBF, L-DED, and WAAM processes can be used for multi-material structures, only powder L-DED offers the required flexibility for the in situ modification of the feedstock composition as an additional process variable [25].

## 2. Additive Manufacturing of Multi-Material Structures

Conventional manufacturing processes cannot yield multi-material components in a single operation. Indeed, single-material parts must be separately produced and joined in a latter fabrication step to create a composite assembly. Conversely, multi-material AM unlocks the processing of composite structures with either a graded or sharp transition between materials, in a continuous step and in a single machine [26]. Hence, the manufacturing chain can be significantly shortened and simplified.

Furthermore, multi-material structures allow for locally tailored material properties. In this manner, different functionalities can be integrated into a single component and the performance can be subsequently enhanced [58]. Moreover, multi-material structures build on the strengths of each of their constituents [59]; thus, they outperform monolithic or single-material solutions. In this manner, a new material-centric fabrication paradigm was made available, characterised by point-wise control of the composition and the material structure [60].

Since the inception of AM, there has been a strong focus on free-form ability and design freedom in terms of geometry [61]. In contrast, material-wise design freedom has gained momentum lately. For instance, the optimisation of the material distribution and the composition to maximise its utilisation has been investigated [62,63]. The emerging research in the multi-material AM field includes the processing of functionally graded structures, bimetallic structures, or metal–ceramic composite materials [64,65,66].

**Figure 8:**
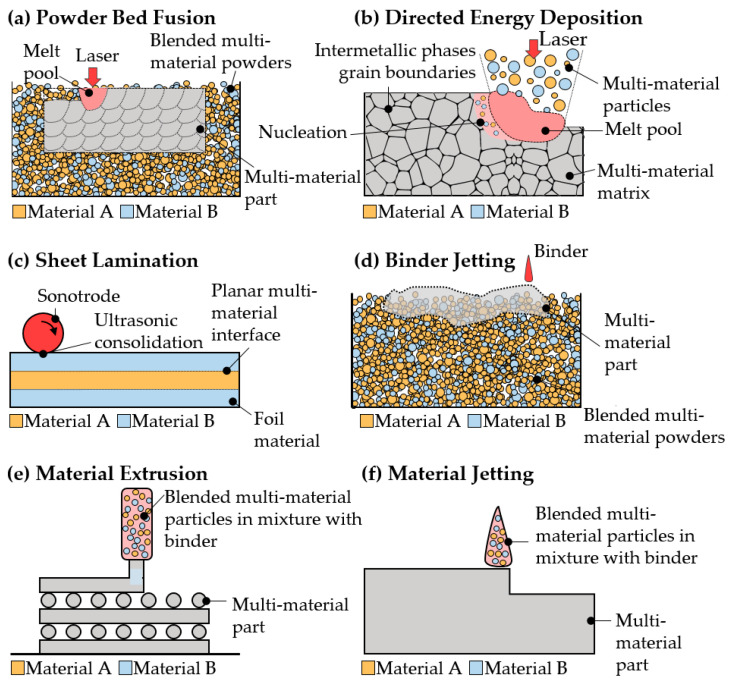
AM process categories suitable for multi-material fabrication. Adapted from [67].

Within the seven AM process categories covered by the ISO/ASTM standard [1], five of them have been identified as having potential viability for multi-material manufacturing [26]. According to Bandyopadhyay and Heer, binder jetting, material jetting, material extrusion, directed energy deposition, and sheet lamination processes have an inherent ability for multi-material AM. However, recent studies have also investigated multi-material powder bed fusion processes, meaning that the multi-material ability of AM processes is still being explored. In Figure 8, the mechanisms of multi-material manufacturing using different AM processes are depicted. In terms of the multi-material ability, a decisive differentiation must be made. Indeed, all of the AM processes mentioned can process mixed materials. However, just a few AM processes can control the material composition locally, meaning that the feedstock composition can be thought of as an additional process variable, which can be adjusted during the build-up. This distinction is made in Table 3.

Although many AM processes are potentially valid for multi-material fabrication, powder-blown L-DED is considered to have a higher potential for heterogeneous multi-material structures, as it is rather easy to modify the composition of the feedstock during the deposition process [36]. Indeed, a unique feature of the L-DED process is its ability for the in situ production of multi-material structures [68,69]. Such is the superiority of L-DED in this regard that the ASTM reported its multi-material ability in their recently developed standards [70]. Among the various advantages of this technology, the ability to process multi-material structures, composites, and functionally graded materials was also listed. Consequently, L-DED is undoubtedly the prevailing technology for multi-material AM.

The tight control of the chemical composition of the melt pool is the key feature, which enables multi-material fabrication via L-DED [62]. In fact, the L-DED system can be designed towards multi-material manufacturing. For instance, different powder materials can be premixed prior to the L-DED process [61]. If higher flexibility is needed, multiple hoppers can be employed to facilitate the in situ modification of the composition of the feedstock [22]. By individually adjusting the powder mass flow rate of each constituent, the composition of the melt pool can by precisely tuned [71]. This latter solution is much more versatile, as the composition of the feedstock can be modified at any time during fabrication [72]. Hence, the composition of the deposited material can be easily adjusted to target the material properties required in each region, wherever a specific functionality is desirable. Commercial solutions with up to 16 synchronised hoppers have already been proposed, with the aim of finely controlling the composition of the fed material [59].

On the other hand, on account of the rapid solidification and high cooling rates of L-DED, the non-equilibrium synthesis of novel materials can be accomplished. Hence, the scope of the possible material systems and structures that are fabricable is further broadened [73]. Consequently, L-DED is being uplifted as a result of its increased material-wise design freedom [74].

In short, the multi-material ability of L-DED unlocks several opportunities concerning the development of advanced materials [8,69,75]. Nowadays, the most relevant multi-material L-DED applications include (i) alloy design, (ii) the processing of high-performance composites, and (iii) functionally graded material manufacturing [74]. Driven by the motivation of this literature review and the advantageous position of L-DED for processing complex multi-material structures, the next sections focus on this process.

### Main Applications of Multi-Material Laser-Directed Energy Deposition

Although most existing alloys were developed decades ago, they are still the preferred solution in the industry. The prevalence of legacy alloys is due to their reliability and suitability for specific applications [59], but also because developing new alloys is extremely resource-consuming. In this regard, the additive-based alloy design approach is substantially cheaper and faster, which boosts the development of new alloys with enhanced performance.

The prospect of outperforming legacy alloys and pure metals is the driver for innovation in alloy engineering. Such is the case for high-entropy alloys (HEAs), which were recently developed [76]. The concept of HEAs involves a design based on multiple elements in equimolar or near-equimolar ratios [77]. As a result, the possible combinations for the alloy design are exponentially increased [22]. It has been reported that HEAs are capable of providing a distinct combination of properties including high strength, hardness, wear, and corrosion or thermal resistance [78]. Most popular HEA materials mainly involve Al, Co, Cr, Fe, Mn, and Ni elements [79], although HEAs combining refractory metals, e.g., Nb, W, or Ta, and other transition metals, e.g., Ti, have also been explored [80,81,82]. Just recently, scientists of the Lawrence Berkeley National Laboratory and the Oak Ridge National Laboratory discovered the toughest alloy known so far, which is, interestingly enough, a CrCoNi HEA [83]. Needless to say, alloy design is now a new R&D topic of AM. Whilst still in its infancy, it is a solution to address the ever-changing demands in materials science.

The production of metal–ceramic composite materials by L-DED has been thoroughly investigated too. This matter involves an inherent difficulty with regard to metal–ceramic interactions. Indeed, a good bond between the constituents must be sought, whilst macroscopically distinct phases should remain. Therefore, the tight control of the energy input needs to be guaranteed to produce a hybrid multi-material system [59]. In this regard, L-DED is a promising solution, as the thermal cycle during the manufacturing process can be precisely controlled while tuning the composition of the feedstock at the same time. The deposition of ceramic particle-reinforced metal matrix composite (MMC) coatings is a prominent application in this field [22]. Various objectives motivate the development of composite materials, including lightening the weight of components without compromising their mechanical capabilities [84], extending their lifespan [85], or enabling the safe operation of components exposed to aggressive environmental conditions, such as high temperatures or corrosive atmospheres [86].

**Figure 9:**
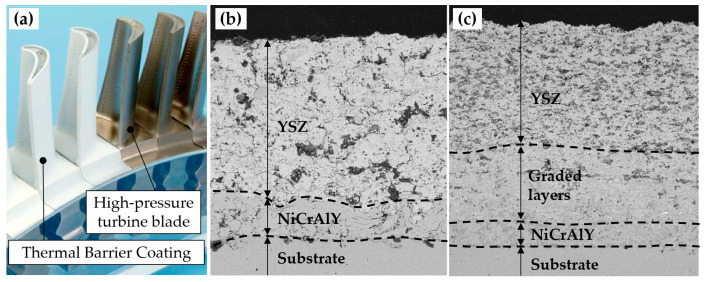
Functionally graded thermal barrier coatings (TBC): (**a**) a high-pressure turbine blade (TBC), courtesy of the German Aerospace Centre (DLR), Institute of Materials Research; (**b**) a conventional TBC; (**c**) a functionally graded TBC. Adapted from [91,92].

Much attention has been also devoted to the development of functionally graded materials (FGMs). The concept of FGM was first devised in 1987 for aerospace applications [87]. FGMs are characterised by having a gradual variation of the microstructure, which results in gradient properties. These kinds of advanced materials can be used to overcome challenging material combinations, such as transitions between incompatible or immiscible alloys. To this end, compositional gradients can be designed to avoid the formation of intermetallic phases or brittle microstructures [88]. Furthermore, site-specific tailored material properties can be attained by using FGMs [65,89]. Lastly, FGMs also facilitate the fabrication of multi-material structures. Indeed, the sharp material transitions can be substituted by gradual interfaces. Hence, the stress concentrations present in sharp transitions can be avoided [90], while still achieving a good bonding strength in the diffuse interface [72]. For instance, eliminating the sharp transitions in thermal barrier coatings (TBCs) results in the enhanced mechanical performance of high-pressure turbine blades (Figure 9). The production of FGMs through conventional methods is hardly achievable. Fortunately, the multi-material ability of AM processes, and more specifically the fine control and dynamic tuning of the composition of the feedstock in the L-DED process, have enabled the deposition of graded materials. Consequently, the maturation of L-DED processes has brought the focus back to FGMs.

**Figure 10:**
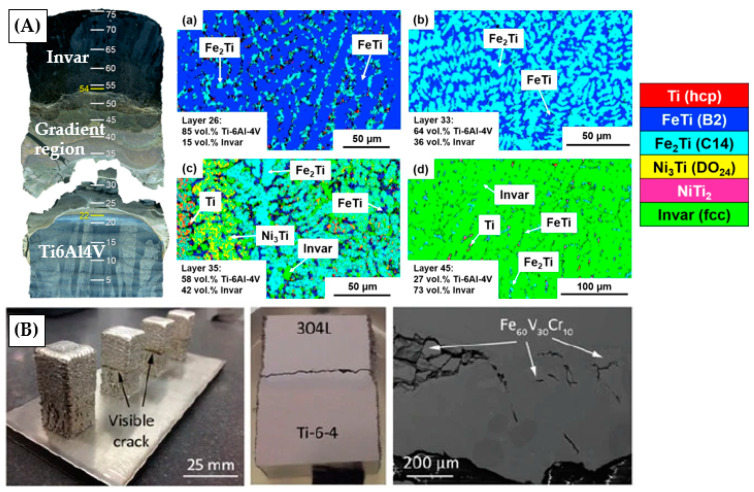
(**A**) Macroscopic cracking in FGM from Ti6Al4V to Invar, promoted by the formation of FeTi and Fe_2_Ti intermetallics, identified by EBSD (reproduced from [93]). (**B**) Macroscopic cracks in FGM from Ti6Al4V to 304L steel as a result of the formation of Fe-V-Cr intermetallics, as evidenced in SEM observations (reproduced from [94]).

Although promising, the field of multi-material L-DED is still in its infancy and many issues need to be addressed before this solution reaches a mature stage. While some challenges are related to the L-DED process itself, additional issues due to the multi-material character of these components need to be faced. The most relevant defects associated with multi-material L-DED are reported in Table 4. Moreover, the origin of such defects and some mitigation strategies are also summarised. In Figure 10, two examples of cracking due to the formation of hard intermetallic phases in multi-material structures are shown.

In this review, the focus is placed on metal matrix composite coatings and functionally graded material deposition, given their industrial relevance and suitability for wear-resistant coating applications. Nonetheless, the reader is referred to previous studies for comprehensive literature reviews on alloy design using L-DED [59,95] and the production of high-entropy alloys (HEAs) [96,97].

## 3. Laser-Directed Energy Deposition of Metal Matrix Composites

**Figure 11:**
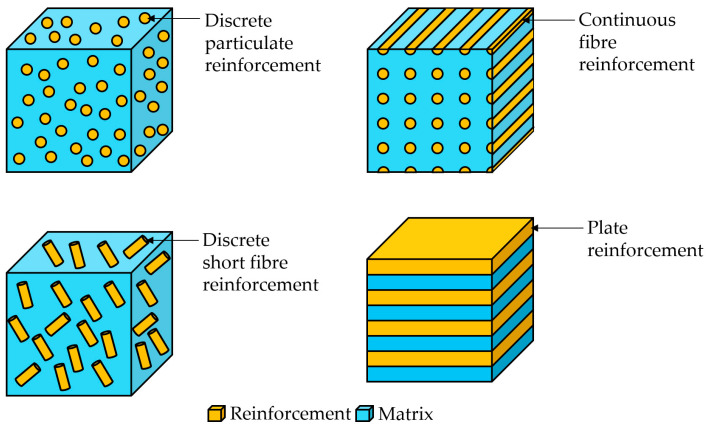
Types of reinforcements in composite materials. Adapted from [102].

Composite materials are defined as those hybrid materials consisting of two or more materials, with clearly distinct interfaces between the constituents. More specifically, the term composite is restricted to those materials that are composed of a continuous matrix, which binds together the discrete phases corresponding to the reinforcement constituent [98]. Different types of reinforcements can be employed to constitute composite materials, namely discrete and continuous (Figure 11). The final properties of the composite materials differ substantially from the properties of their constituents [99,100]. Indeed, the final properties of the composite materials are derived from the individual attributes of the constituents [101].

### 3.1. Origin of Metal Matrix Composites

Metal matrix composites (MMC) refer to those composites constituted by a metallic matrix and typically reinforced by a ceramic phase [103]. In MMC material systems, the metallic phase serves as a binder to the composite, while the ceramic phase acts as the reinforcement. MMCs emerged in the 1960s to further improve the mechanical properties of structural superalloys for applications related to defence and aerospace [104,105]. Later, in 1991, the suitability of these materials for the surface modification of components exposed to critical erosion and wear conditions was established [106]. At that time, the synthesis of MMCs was barely possible, which is why not as many extensive and comprehensive studies have been carried out in this field. However, owing to the rapid development of AM and the recent popularity of multi-material AM, the research interest in MMCs has risen accordingly.

In terms of the industrial requirements, the development of MMCs has been driven by the need for complex materials with a higher hardness to produce wear-resistant coatings for high-end applications [68,107]. This problem has been traditionally addressed through alloy design, i.e., by developing adequate alloys to meet the requirements set by the industry. In this regard, hardfacing with Fe-, Ni-, and Co-based alloys using L-DED for wear- and corrosion-resistant coating applications have been broadly investigated [108,109,110]. Furthermore, alloying with elements such as niobium, vanadium, and tungsten has been demonstrated to extend the tooling lifetime [111]. However, every so often, monolithic materials fail to provide a suitable solution for industrial problems [112], and when an additional improvement of the wear resistance of metal alloys is required without compromising their toughness, composite materials are typically considered. In fact, in monolithic alloys, increasing the hardness of materials typically comes at the expense of a loss in ductility [113]. Conversely, ceramic-reinforced MMCs yield superior material properties provided by the individual contributions of their constituents, e.g., the high hardness and high strength-to-weight ratio of the ceramic phase, in combination with the high toughness and ductility of the metal matrix [114]. Therefore, surface engineers often resort to ceramic hard metal coatings to increase the resistance to wear [115]. Fortunately, laser processing technologies enable a localised dispersion of ceramic particles [116] or the deposition of ceramic particle-reinforced MMC coatings [117].

### 3.2. Advantages and Applications of Metal Matrix Composites

MMCs benefit from the high hardness, strength, and wear resistance of the ceramic phase, but also from the high ductility and good electric and thermal properties of the metallic phase [59]. Overall, ceramic-reinforced MMCs have been proven to offer superior properties in terms of their strength, hardness, wear, and corrosion resistance, and also to behave well even when exposed to high-temperature conditions [118]. The reinforcement phase can strengthen the metal binder in many ways, with coating applications being in the spotlight [26]. On the one hand, when the composite material is subjected to an external load, it is transferred from the metal matrix to the ceramic particles in an efficient manner [119]. On the other hand, the discrete particles inhibit the movement of dislocations and restrict plastic deformation. As a consequence, the metallic phase is retained and the wearing out is prevented [118]. This is the reason behind the higher wear resistance of MMCs over monolithic alloys [120].

**Figure 12:**
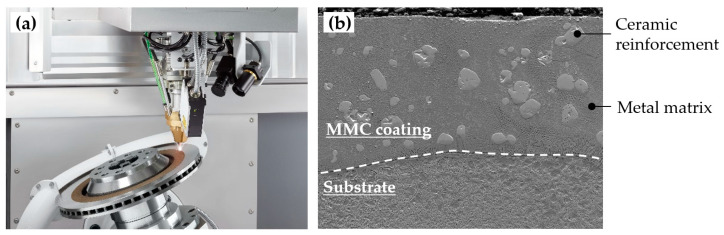
(**a**) MMC coating deposition on a brake disk (reproduced from [129]) and (**b**) wear-resistant MMC coatings, adapted from [130].

Owing to the outstanding behaviour of MMCs, they have been widely proposed to improve the surface properties of highly demanded components, as an alternative to conventional metallic alloy systems [121]. They are of particular interest in those applications where conventional alloys lack sufficient hardness and wear resistance [122], such as in aerospace [73], die and mould [123], agricultural [124,125], mining [126], automotive [127], and oil and gas applications [128]. In Figure 12a, an application example of MMC coatings is shown, involving the wear-resistant coating of a brake disk. In short, they are an interesting solution for those applications where wear, erosion, and corrosion are the main mechanisms responsible for the failure of components [69].

### 3.3. Production of Metal Matrix Composites

Back when the potential of MMCs was barely glimpsed, their production was strongly limited by the processing techniques available at the time, as their manufacture by conventional methods is complicated [118]. The low ductility and low fracture toughness are extremely challenging and limit their processability using casting or powder metallurgy techniques [22]. In the last decade, and owing to the rise of AM technologies, namely L-DED, a significant effort has been invested into researching this topic. According to Bandyopadhyay et al., if the challenges related to the production of MMCs are overcome, high-performance components with good behaviour could certainly be attained [26]. Despite the production of these material systems remaining problematic, according to Mostafei et al., L-DED is a reliable method for manufacturing MMCs, and it is particularly suitable for the deposition of MMC coatings [131]. As a matter of fact, the high flexibility in terms of the materials and the tight control of the composition of the multi-material mixtures place L-DED in a highly competitive position for the production of MMC parts and coatings [68,132].

**Figure 13:**
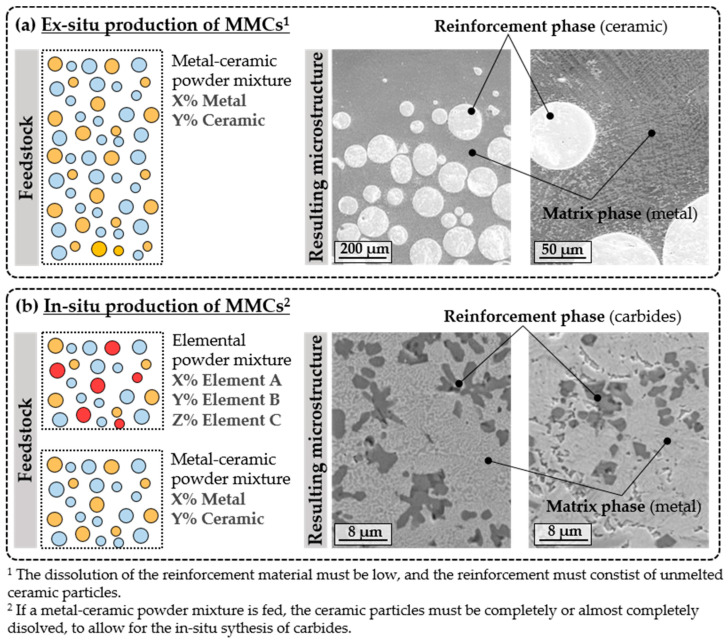
Formation mechanisms of MMCs using L-DED. Images from [134,135].

MMC structures are typically fabricated through powder-based processes, where the ceramic and the metallic powders are premixed in ratios ranging from 1 to 20 wt. % [133]. Nonetheless, mixtures containing higher amounts of the ceramic phase are also frequently reported. The L-DED of MMCs can be approached in two different ways, depending on the mechanism for the formation of the reinforcement phase (Figure 13) [22].

1. Ex situ production of MMC (Figure 13a): The first approach consists of the projection of a powder mixture with a precise volumetric fraction of the ceramic phase into the melt pool. In either case, the reaction between the ceramic and the metallic phase is limited and controlled. To this end, the process parameters and the feedstock morphology and granulometry should be selected so as to guarantee that no excessive dissolution of the ceramic phase occurs. In addition, the process parameters should be selected so as to guarantee proper bonding between phases.
2. In situ production of MMC Figure 13b): In the second approach, a mixture of elemental powders is introduced into the melt pool. The high processing temperatures used in L-DED allow chemical reactions between elements to occur, resulting in the formation of disperse carbides or intermetallics. Conversely, a ceramic–metallic powder mixture can be fed but the complete decomposition of the ceramic phase must be ensured so that the in situ synthesis of dispersed carbides takes place. In this manner, MMCs may be in-situ-synthesised. In both cases, the process parameters and the powder morphology should be carefully selected to facilitate the in-situ synthesis of carbides.

**Figure 14:**
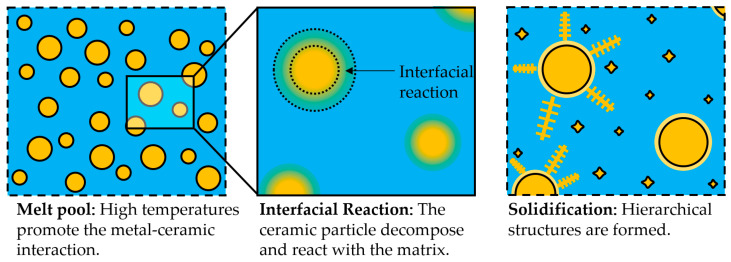
Schematic illustration of the metal–ceramic interaction during the L-DED of MMC coatings and formation of hierarchical structures.

According to the formal definition of the composites, the material systems obtained through the second approach do not correspond to MMCs but rather to alloys with dispersed hard phases, as no macroscopic phases could be distinguished. However, these complex alloys are often referred to as MMCs in the literature. In addition, controlling the dissolution of the ceramic phase in ex-situ-produced MMCs is not a trivial matter. In ceramic-reinforced MMCs produced via L-DED, the ceramic particles often suffer partial dissolution, which promotes interfacial reactions between the ceramic and metallic materials, as shown in Figure 14 [136]. As a result, complex hierarchical structures are often observed. This phenomenon has been investigated in the literature; hence, the factors governing it and its consequences are more extensively discussed in Section 3.4.

In essence, L-DED is a highly competitive technology that has the potential to form MMC structures, owing to the high flexibility when processing the multi-material structures [137]. At the same time, L-DED enables the deposition of high-quality surface coatings, which is the main application of ceramic-reinforced MMCs. Driven by this motivation, several aspects of the production of MMCs through L-DED have been investigated. Accordingly, the most relevant studies in the literature are summarised.

### 3.4. Most Relevant Literature on L-DED of Metal Matrix Composites

**Figure 15:**
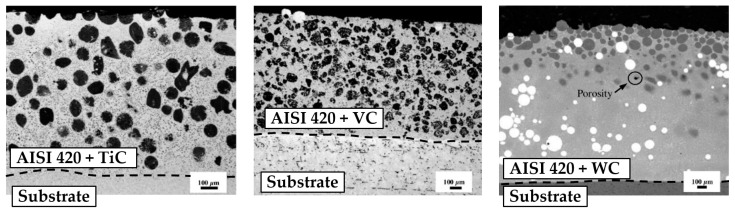
MMC coatings constituted by an AISI 420 matrix and different reinforcement materials. Adapted from [128].

The research carried out in this field has focused on the analysis of different material systems and their performance. For instance, Nurminen et al. compared various material systems consisting of a metal binder and disperse carbides [138]. They focused on addressing material compatibility issues and their effect on the performance from a tribological perspective. They concluded that the properties of MMCs were strongly linked to the material selection and the chemical affinity between the constituents. Indeed, material systems with high affinity would promote the decomposition of the ceramic phase, which eventually was found to be detrimental to the performance of the coating. Jiang and Kovacevic fabricated MMC coatings containing TiC and AISI H13 tool steel and compared the tribological behaviour of this material system to other material systems previously reported in the literature [139]. Adam et al. investigated the performances of different material systems for ballistic applications and studied the suitability of L-DED for the production of MMC coatings [140]. Zhang and Kovacevic investigated the tribological performance of MMC coatings composed of an AISI 420 steel matrix and different carbides with the aim of providing some insights on material selection [128] (Figure 15). These studies demonstrated the adequacy of MMC coatings manufactured by means of L-DED for the surface modification of components exposed to severe operating conditions and outlined some guidelines to facilitate material selection. However, a limited focus was placed on understanding how the processing conditions affect the performance of MMC coatings within a sole material system. Hence, generic conclusions may be drawn, but no deep understanding was gained concerning the specific microstructures that should be targeted and no methodologies were proposed to materialise it.

**Figure 16:**
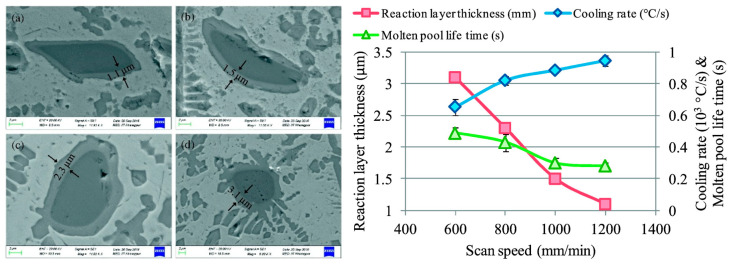
Reaction layer and thickness of the reinforcement phases of MMC coatings with varying melt pool lifetimes and variations with respect to the scan speed. Reproduced from [143].

Other studies focused on the microstructural evolution of MMC coatings due to the high-temperature processing of L-DED. As mentioned above, the dissolution of the ceramic particles in ex-situ-produced MMCs is hardly avoidable, and this phenomenon affects both the microstructural aspects and mechanical properties. Li et al. investigated (Cr, W)_23_C_6_- and WC-reinforced Fe-based composite coatings. They reported the phase transformations occurring as a result of the L-DED process. Disperse eutectic carbides were found in the matrix structure, but fine carbides also precipitated in the interdendritic phase [122]. Along the same line, Zhao et al. investigated the dissolution of the reinforcement phase for WC-Ni material systems in [141] and WC-Fe in [142]. In their studies, a thorough characterisation process of the microstructure generated as a result of the interaction between the reinforcement phase and the matrix phase was carried out. They observed that carbides with different morphologies were dispersed in the metal matrix, while retained particles were also encountered. In addition, the main mechanisms responsible for the decomposition of the ceramic particles were reported, namely dissolution, diffusion, partial or complete fragmentation, and precipitation. From a processing perspective, Muvvala et al. investigated the metal–ceramic interface in a WC-reinforced Ni-based alloy. They found that the decomposition of the reinforcement particles played a major role in the bonding type. Moreover, they measured the melt pool lifetime and correlated it with the thickness of the reaction layer surrounding the ceramic particles, as shown in Figure 16 [126].

These studies have brought to light the importance of the interactions between the constituent phases in MMC composites, especially considering the high temperatures inherent to L-DED processing. However, few studies have been carried out involving a thorough investigation and discussion of the effects of the process parameters and the thermal cycle of the manufacturing process. In fact, none of the previous authors found a correlation between the process parameters, microstructure, and resulting properties of the coatings. Hence, the role of the processing parameters in the metal–ceramic interactions and the microstructures and properties of MMC coatings has been neglected, especially in the formation of unexpected microstructural phases. It is out of the question that the L-DED parameters are responsible for the thermal cycle generated during processing. Thus, the process parameters affect the dissolution of the reinforcement particles, and presumably the mechanical properties, such as the hardness, of MMC coatings.

In this line, another aspect that has attracted the attention of researchers is the hardness of MMC coatings and the correlation between the hardness and the volumetric fraction of the reinforcement phase. Most authors report that increasing the volumetric fraction of the reinforcement phase results in a substantial enhancement of the hardness. Li et al. observed a gradual increase in hardness with increasing volumetric fractions of WC from 0% to 20%. They ascribed this to the formation of carbides and the solid–solution strengthening due to lattice distortion [122]. Xie et al. and Raahgini et al. reported a nearly linear correlation between the hardness and the volumetric fraction of the reinforcement phase for Co-WC [144] and Ni-VC [120] material systems, respectively. Deschuytenner et al., on the other hand, characterised the multi-scale hardness of a WC-reinforced Ni-based alloy. In short, they studied the normalised hardness of the composite samples, but also the hardness achieved by the metal matrix as a result of the microstructural modification [145]. Ostolaza et al. investigated the effect of the processing parameters and the feedstock composition on the hardness of the matrix and on the hardness of the composite in WC-Co coatings through statistical regression methods [146]. It was concluded that longer interaction times promoted the decomposition of the ceramic particles, which resulted in an increase in the W content in the matrix. At the same time, different mechanisms responsible for the hardening of the MMC coatings were reported, namely grain refinement and carbide precipitation, depending on the feedstock composition and processing conditions. Zhao et al., on the other hand, quantified the loss in hardness when the dilution of the substrate increased [141]. As expected, the higher the amount of substrate material that was melted, the lower the hardness of the coating material. Ultimately, having higher substrate dilution alters the composition of the coating. If the substrate material is softer than the coating material, then the overall hardness of the coating will decrease.

**Figure 17:**
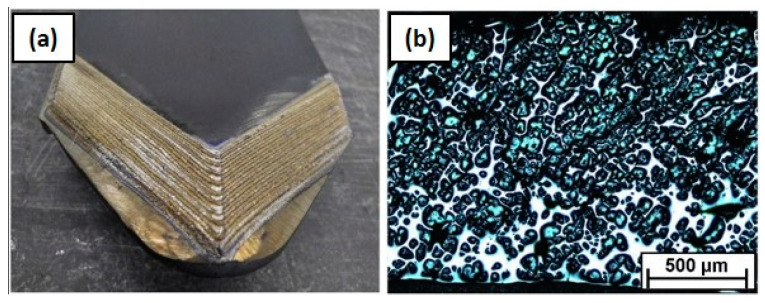
(**a**) An agricultural tool coated with Stellite 6 and WC and (**b**) microstructure of the applied coating. Reproduced from [125].

Lastly, many authors have tried to demonstrate the suitability of MMCs for high-performance coating production by studying their wear resistance. Li et al. investigated the tribological behaviour of (Cr, W)_23_C_6_-WC-reinforced, Fe-based coatings. They obtained good results in terms of the wear resistance of the MMC samples. More interestingly, they found that the surface properties were maximised for an optimal coating composition [122]. Xie et al. studied the wear resistance of WC-Co MMCs and reported a positive correlation between the wear resistance and the WC content of the MMC [144]. Similar conclusions were reached by Zhao et al. for WC-Fe [142] and WC-Ni [141] MMC coatings. Their experimental results suggested that a significant gain in tribological performance could be obtained by increasing the WC content. They ascribed this effect to two phenomena, namely the retained WC particles that remained unmelted and the dispersed carbides throughout the matrix. Bartkowski and Kinal achieved increased wear resistance by embedding WC particles into a Stellite 6 matrix as compared to the monolithic Stellite 6 coatings [124]. They concluded that the excess WC content or insufficient hardness of the matrix could promote a more intensive wear mechanism in MMC coatings. In a later publication, they obtained promising results when testing the behaviour of the designed coating in real conditions when applied to an agricultural tool (Figure 17). They reported a 25% increase in the life of the tooling [125].

In this regard, Muvvala et al. carried out a comprehensive investigation on the contribution of the ceramic phase to wear resistance [126]. They concluded that the uncontrolled dissolution of the ceramic particles caused the embrittlement of the matrix phase. As a result, the fracturing and spalling of the surface during wear testing were promoted; hence, these samples suffered a higher wear rate. It can be concluded that increasing the reinforcement content does contribute to a higher wear resistance of MMC coatings, provided that the ceramic particles do not fracture and that the third body mechanism is avoided [120]. Similar conclusions were previously reached by Fernández et al. [147]. In MMC coatings, the discrete reinforcement particles rule the plastic flow of the metal matrix. Hence, increasing the reinforcement phase changes the main wear mechanism; indeed, it switches from severely adhesive to mildly adhesive and abrasive wear [148].

The high-temperature wear resistance of Co- and Ni-based MMC coatings has also been investigated. Erfanmanesh et al. studied the high-temperature tribological behaviour of WC-reinforced Ni- and Co-based matrices [149]. The reinforced samples showed superior wear resistance and they found that soft abrasive and adhesive mechanisms were responsible for the wear damage. Wang et al. also tested the high-temperature wear resistance of WC-Co MMC coatings and confirmed the previously reported good behaviour of this material system [123]. In addition, the experimental results showed a positive correlation between the wear resistance with respect to the WC content. However, they reported a significant drop in the thermal fatigue life when increasing the WC content over 20% wt. Lastly, Karmakar et al. investigated the abrasive wear of WC-Co- and Co-coated AISI H13 tool steel substrates at high temperatures [109]. The ceramic-reinforced coatings yielded greater resistance to wear up to 650 °C as compared to the un-coated substrates. The superior surface properties of the reinforced coatings were more evident the higher the temperature of the abrasive test.

Although the behaviour of MMC coatings in terms of wear has been thoroughly studied, other aspects that tightly limit their performance have been completely neglected. For instance, little effort has been devoted to the evaluation of the interfacial strength between the MMC coating and the substrate, which will directly affect the durability of the surface-treated components. Indeed, the flexural strength of coated parts and the residual stresses induced during processing are key aspects that limit the life and the safe operation of coated components [150]. It is without question that the surface properties and mechanical behaviour of MMC coatings are ruled by the strength of the interfacial bonds between the macro-constituents, as has been stated by several researchers [151,152]. On the one hand, the reaction layer guarantees a metallurgical bond; therefore, the cohesion of the composite is ensured and one should expect proper load transfer between the macroscopic constituents [152]. Conversely, the dispersion of discrete carbides throughout the matrix may cause a loss in ductility, which is detrimental to the structural behaviour of the composites [153]. Thus, the key lies in finding a balance in the extent of the metal–ceramic interaction. Metallurgical bonding between the matrix and the reinforcement should be sought, but the embrittlement of the metal matrix should be prevented to preserve the good mechanical properties of the matrix. This issue may be solved by optimising the process parameters and by tuning the thermal cycle during processing [126].

In Table 5, a summary of the most relevant publications regarding the L-DED of MMC coatings is provided.

In terms of the processability of MMCs through L-DED, the realisation of good-quality coatings is still a challenge. For instance, material incompatibility is an issue that needs to be tackled [36]. Moreover, defects related to the metallurgical integrity (pores or cracking) are frequently encountered [131].

The cracking of MMCs deposited by L-DED has been widely studied in the literature [118]. Cracks in these coatings originate from the high residual stresses generated during the deposition process [131]. As a process based on the fusion and rapid solidification of materials, parts manufactured by L-DED withstand high temperature gradients; thus, high thermal stresses can be produced if specific care is not taken [118]. In addition, the interaction between the particles and the matrix tends to provoke matrix embrittlement. The loss of ductility prevents the metal matrix from absorbing the residual stresses of the manufacturing process. Additionally, the reinforcement particles act as stress concentrators [109], which further exacerbate cracking. Consequently, it has been stated in the literature that these materials often suffer not only from cracking phenomena but also other sorts of catastrophic failures due to high residual stresses and material property mismatches, such as delamination [154].

Multiple methods have been proposed to eliminate cracking, namely substrate preheating, the addition of rare earth oxides, or the elimination of the sharp transition between substrates and coatings through the use of functionally graded materials (FGMs) [117]. Driven by this requirement, in Section 4, the origin of FGM structures is described and the implementation of the FGM strategy for the fabrication of high-quality MMCs is explored.

## 4. Laser-Directed Energy Deposition of Functionally Graded Materials

### 4.1. Origin and Definition of Functionally Graded Materials

**Figure 18:**
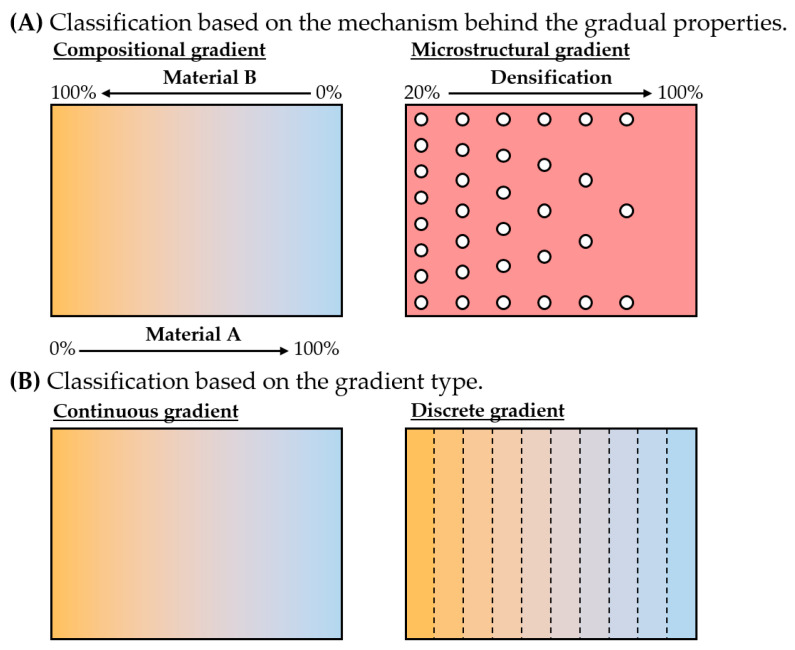
FGM types according to different classification criteria: (**A**) mechanism of the gradual properties and (**B**) the gradient types.

In short terms, FGM materials are a class of advanced materials in which spatial variation of the material properties occurs within a single structure [155]. Different criteria can be established to differentiate FGM structures. One could classify FGMs based on the method followed to obtain such variable properties. Indeed, variable material properties can be achieved through compositional or microstructural modifications [62] (Figure 18A). Conversely, a classification criterion based on the gradient type has also been proposed, i.e., discrete and continuous gradients [22] (Figure 18B). Either way, location-dependent material properties without sharp transitions can be attained using FGM structures [155].

The first record of functionally graded materials (FGM) corresponds to the development of this concept to overcome the limitation of conventional composite materials in thermal barrier coatings (TBC) for aerospace applications [87,156]. As a matter of fact, the discovery was driven by the requirement of a TBC that would withstand a temperature gradient higher than 1000 K [157]. Before coming up with the FGM concept, conventional laminate composites were tested with little success. It was observed that the sharp interface was responsible for the failure of the composites. Owing to the difference in the CTEs between the metal and ceramic constituents, the conventional composites were unable to endure the high thermal gradients [36,62]. Nowadays, FGMs are present in many other applications, as a tool for tailoring mechanical, thermal, or electrical properties in a location-dependent manner within one sole component [22,26].

**Figure 19:**
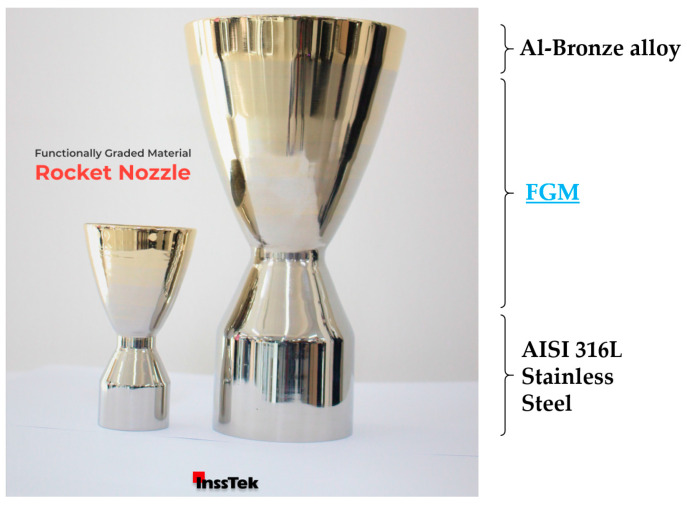
The FGM rocket nozzle, a case study by InssTek (adapted from [158]).

In Figure 19, an FGM rocket nozzle is shown, which was developed by InssTek as a case study to demonstrate the capability of L-DED to build AISI 316L to Al-bronze FGM components at real industrial scales.

### 4.2. Production of Functionally Graded Materials

The low processability of FGM structures through conventional manufacturing methods has limited their development to date. FGMs can certainly be produced through vapour deposition, powder metallurgy, or centrifugal casting [62]. However, these processes have little flexibility and are resource- and time-consuming. AM processes, on the other hand, have demonstrated their suitability for the production of FGM structures [159]. Indeed, the PBF technology can be used to achieve a layer-wise variation of the microstructure of a single material by modifying the grain orientation [160], via the controlled vaporisation of certain elements [161], or via the controlled modification of the porosity [162]. In contrast, compositional gradients can be attained using DED owing to the unprecedented design freedom and multi-material ability [22]. Many authors agree that L-DED is the preferred solution for the fabrication of FGM structures [22,36,62], which is only logical, considering that the production of FGM structures requires tight control of the chemical composition and the flexibility to fabricate multi-material components, while allowing the modification of the feedstock composition within one single build-up [163].

In addition to the potential for the production of location-dependant material properties, FGMs have been proven beneficial in mitigating residual stresses [164] or substituting sharp interfaces, with a high risk of delamination failure [22]. In terms of the operational behaviour, sharp material transitions are considered stress concentration zones and complex loading conditions may cause a catastrophic failure.

### 4.3. The Most Relevant Literature on L-DED of Functionally Graded Materials

**Figure 20:**
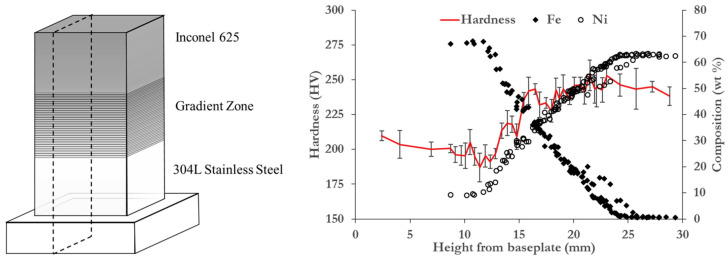
Additively manufactured FGM from 304L stainless steel to Inconel 625 Ni-based alloy and evolution of the Fe and Ni elemental contents as opposed to the hardness. Reproduced from [167].

Driven by the benefits of FGM structures, much research has been carried out with the aim of attaining a controlled grading between different metal alloys. In particular, gradients between Ni-based alloys and different grades of stainless steels have been extensively reported, due to the interest from the power generation and aerospace industries in this material system. For instance, Zhang et al. studied the microstructure, hardness, and tensile fracture values of AISI 316L to Inconel 625 functionally graded structures. Firstly, they observed a hardness gradient along the graded region, which matched the microstructure. Additionally, the fracture in the tensile testing occurred in the AISI 316L section, while the interfaces survived the testing owing to the graded transition [165]. Su et al. studied the microstructure of AISI 316L to Inconel 718 graded samples and found that austenitic formation was promoted in the graded region, leading to the loss of hardness in certain areas [166]. Carrol et al. analysed AISI 304L to Inconel 625 gradient samples, and they found cracks of several microns due to the formation of carbides in the graded region [167]. In Figure 20, the compositional design of the sample built by Carroll et al. and the evolution of the Fe and Ni elemental contents in contrast with the variation in hardness values are shown. Li et al. just recently developed a multi-physics model to better understand the governing phenomena in the L-DED of 316L to Inconel 718 FGM structures. They found that the composition of the feedstock substantially affected the melt pool size as a result of the differing thermal properties. Indeed, increasing the Inconel 718 content reduced the heat dissipation and resulted in a larger melt pool. Moreover, they found that layers that had the same nominal composition contained a non-uniform distribution of Inconel 718, which they attributed to the varying melt pool sizes and thermal gradients [168].

In contrast, other material combinations and systems have been studied too. In this regard, Fan et al. investigated Invar to MnCu FGM samples. They reported a non-linear variation of the hardness values, which was attributed to the solid–solution strengthening mechanism being promoted in the gradient region, where the highest hardness was measured. Moreover, they studied the tensile behaviour of the graded structures and found that the deformation behaviour was not homogeneous due to the stress concentrations. They concluded that the strain was directly correlated to the hardness evolution, and that the softer regions contributed the most strain [169]. In contrast, Ji et al. fabricated Ti6Al4V to Inconel 718 graded coatings and they compared the expected microstructures and phase transformations based on computationally derived phase diagrams and experimental observations. They concluded that the Ti-Ni combination resulted in the generation of Ti_2_Ni and TiNi intermetallics, based on both the experimental and computational results. Moreover, they studied the high-temperature behaviour of these materials and they found that the diffusion zone between the graded layers increased with increasing exposure to high temperatures [170]. In addition, Nam et al. compared the deposition of AISI 316 onto a mild steel substrate with the Fe/AISI 316 gradient deposition and found that while many pores and cracks were observed in the sharp transition, no cracking was detected in the graded sample [171].

Although promising, the production of FGM structures based on multiple ceramic phases is still a challenging matter. Based on the reviewed literature, some critical issues are described herein. The high-temperature processing and mixing behaviour of different alloys highlight the need to consider metallurgical aspects. Indeed, the correlation between the composition, microstructure, and mechanical properties is not linear, especially when phase transformations and intermetallic formation occur. Therefore, from a material science perspective, a computational approach appears to be the most efficient method to anticipate such issues and to design the FGM structure accordingly. From the processing perspective, the effects of the feedstock composition on the thermal aspects of the process need to be further understood. Indeed, it has been demonstrated that the differences in the thermal properties of the graded alloys significantly affect the formation of the melt pool and the thermal history of the process. In this regard, Zhang et al. reported instability and shaking of the melt pool during the deposition of 316L onto Inconel 718, which happened only in certain compositions. They proposed that this effect could be governed by the different inertial properties of the powders employed to fabricate the sample [172]. However, more research is needed to really understand which phenomena affect the production of FGMs and their roles and significance.

Some publications related to FGM structures have tackled metal–ceramic material systems, and more specifically the fabrication of functionally graded MMCs. For instance, Wang et al. fabricated FGM composites that ranged from 100% Ti6Al4V alloy to 60%Ti6Al4V and 40% Ni-coated graphite. They reported a nearly linear correlation between the hardness and the composition of the powder mixture [173]. On the other hand, Ramakrishnan and Dinda employed the FGM strategy to deposit MMC coatings consisting of Haynes 282 superalloy and SiC [174]. In this regard, Wei et al. also focused on introducing a composition gradient in MMC parts and they manufactured a TiC/Ti6Al4V functionally graded composite, reaching similar conclusions to those previously reported [175].

In Table 6, the most relevant publications on the fabrication of FGM structures through L-DED are summarised.

**Figure 21:**
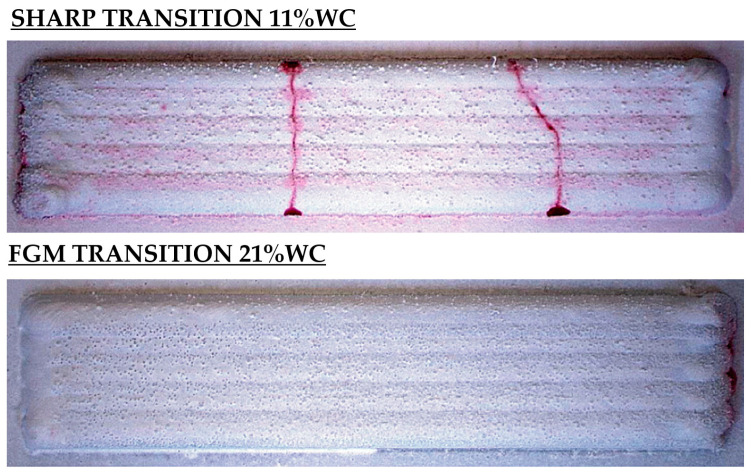
Reduction in crack sensitivity with the FGM strategy in Stellite 6 and WC MMCs. Adapted from [176].

With the aim of exploring the novel applications of FGM structures and going back to the production of MMC coatings, one of the main issues in the L-DED of MMCs is the lack of metallurgical integrity, high crack sensitivity, and delamination, which can be mitigated by implementing an FGM strategy. On this basis, Xu et al. investigated the crack sensitivity of mono-compositional and functionally graded MMC samples. They concluded that functionally graded transitions could significantly contribute to mitigating cracking in MMC coatings, as shown in Figure 21 [176].

**Table 6:** Most relevant publications concerning the L-DED of FGM structures.

Publications	Materials	Application	Main results	Limitations/ Observations
Carrol et al., 2016 [167]	100% AISI 3104L to 100% Inconel 625	Aerospace and nuclear power generation	Cracking at a precise composition was due to the formation of carbides, and CALPHAD simulations were able to predict it	A crack-free sample could be probably fabricated, avoiding the composition where hard carbides are stable and prone to form.
Nam et al., 2018 [171]	100% Fe to 100% 316L	Miscellaneous	Directly depositing 316L onto mild steel resulted in cracking, while the FGM sample had no apparent defects.	No analysis of the evolution of the mechanical properties or behaviour of the samples was provided. FGM samples still showed a significant amount of pores.
Zhang, Chen, and Liou, 2019 [165]	100% AISI 316L to 100% Inconel 625	Die and mould	Defect-free FGM samples were successfully deposited and gradual hardness was observed. The tensile behaviour of the FGM samples was in-between pure AISI 316L and Inconel 625.	It would be interesting to compare the behaviour of the FGM sample to that of a sample having a sharp transition between AISI 316L and Inconel 625.
Su et al., 2020 [166]	100% AISI 316L to 100% Inconel 718	Nuclear power plants and oil refineries	The compositional step to form the gradient affects the hardness and tensile properties of the FGM sample.	The variability in FGMs designed with different discretisation steps was ascribed to the thermal cycle and processing conditions. The actual effect of the FGM design was not properly tested, as samples having different sizes and amounts of layers are used for comparison.
Ostolaza et al., 2021 [177]	100% AISI 316L to 100% AISI H13	Die and mould	The compositional gradient did not guarantee a gradual variation of the material properties, namely the hardness and the corrosion resistance.	The FGM sample shows severe cracking, which is ascribed to the formation of the sigma phase. The CALPHAD methodology could be employed to design an FGM sample in which the formation of such hard phases is minimised.
Wang et al., 2021 [173]	100% Ti6Al4V to 40% graphite 60% Ti6Al4V	Armour, gear, and cutting tools	Ti-Ni-C graded samples showed a gradual hardness and microstructure as a result of in-situ TiCx reinforcement formations.	Further investigations should focus on evaluating the mechanical properties of FGM structures as compared to sharp transitioned samples.

## 5. Conclusions

The ability of L-DED to manufacture multi-material components is one of the most compelling aspects of this technology, and so it has been acknowledged by industry and research bodies. The current challenges related to joining dissimilar materials, for instance, can be overcome through the use of multi-material L-DED, as components composed of different materials can be built up in a single operation. The prevalent applications of multi-material L-DED are alloy design, metal matrix composites, and functionally graded materials.

With respect to alloy design, L-DED permits much faster and more cost-efficient experimental trials when testing new alloys. This is key in the development of new high-entropy alloys, where the possible compositions to be tried out are manifold and simplifying the experimental testing is absolutely necessary. On the other hand, surface engineers can obtain major benefits from metal matrix composite coatings, as they behave outstandingly well in terms of wear. Lastly, the use of functionally graded materials seems to be a key strategy to enable the joining of a priori incompatible material combinations. The straightforward ability of L-DED to control the feedstock composition and modify it as an additional process parameter has provided evidence of the suitability of this process for multi-material fabrication, and it is already considered the prevailing technology for forming multi-material structures. However, some issues need to be addressed before this technology is ready for industrial use. Some of these challenges concern process engineering and materials science, which are summarised below.

In terms of process engineering, the composition of the feedstock injected into the melt pool needs to be tightly controlled. If the L-DED system involves several powder hoppers, then the individual mass flow rate of each one of the hoppers needs to be precisely controlled, and the dynamic behaviour when varying the composition of the feedstock during the build-up should be carefully understood. Downstream, the fluid dynamic behaviour of multi-material powder mixtures needs to be known. Indeed, the concentration of the powder mixture by the nozzle depends on the material properties. This is especially troubling when working with metal–ceramic powder mixtures, as the inertial properties of ceramic and metallic materials differ significantly. Failure to understand and predict the behaviour of the powder feeding system from the powder feeder to the injection of the powder mixture into the melt pool will inevitably cause deviations between the nominal composition and the real one.

Moreover, in view of the impact of the thermal cycle during processing on the properties and integrity of multi-material L-DED, the effect of the process parameters on the thermal history should be investigated. Eventually, the process parameters will be tuned to target a specific thermal cycle. Moreover, in the case of MMCs, the thermal history will be defined and controlled to find a balance in the interactions between the ceramic and the metallic phases. With regard to materials engineering, a deeper understanding should be gained of how the thermal cycle affects the interactions between the constituents in a multi-material powder mixture. Considering that the non-equilibrium synthesis prevails in L-DED, extensive databases should be developed to ease the set-up process and target or avoid specific microstructures. In addition, the in-service behaviour of multi-material structures needs to be tested, whether it be the tribological performance or the structural behaviour. Based on that knowledge, generic guidelines should be drawn so that the material selection and the choice of processing conditions are facilitated.

## Figures and Tables

**Table 2:** L-PBF, L-DED, and WAAM comparison [45,46,47,48,49,50,51,52,53,54,55,56,57].

Feature	L-PBF	L-DED	WAAM
Part dimensions [mm]	max. 600 × 600 × 600	Virtually unlimited	Virtually unlimited
Surface finish, Ra [µm]	9–16	5–30	200
Dimensional accuracy [mm]	0.05–0.1	0.5–1.0	1.0–2.0
Build rate [g·min^−1^]	3–4	6–50	300–400
Densification	>99%	>99%	>99%

**Table 3:** Differentiated multi-material ability of AM processes.

Can Process Mixed Feedstock	Can Control the Composition of the Feedstock Locally
Powder Bed Fusion, Binder Jetting	Directed Energy Deposition, Sheet Lamination ^1^, Material Extrusion, Material Jetting

^1^ The ability of sheet lamination processes to control the feedstock composition locally is restricted to a layer-wise discrete variation.

**Table 4:** Main defects associated with multi-material L-DED [25,26,36].

Defect	Origin of Defect	Proposed Mitigation Strategy
Microstructure and property heterogeneity	The inhomogeneous distribution of the multi-material mixture constituents due to differences in material densities (heavier particles may sink) and the liquid surface tension.	Careful material selection (composition and powder size) and control of the solidification rate (faster solidification) will inhibit heavy particles from sinking.
Selective vaporisation of elements	Differences in thermal properties (i.e., thermal conductivity, melting temperature) and laser absorptivity levels of constituents make the distribution of the heat input challenging, and there is a risk of causing preferential vaporisation of low-melting elements.	Careful control of the thermal cycle of the process and adjustment of the mixture composition to account for this vaporisation preventively.
Deviation from target multi- material composition	Differences in the inertial properties of the multi-material feedstock constituents (i.e., density and powder granulometry) may cause in-flight segregation of the materials during the injection. If materials are concentrated differently by the nozzle, the composition of the powder mixture entering the melt pool might differ from that being fed by the powder feeder.	New nozzle concepts, where the design agrees with the powder flow behaviour of each material. Conversely, if the concentration of the powder can be anticipated, the concentration of the powder provided by the powder feeder can be modified to target the nominal composition in the melt pool.
Cracking–alloy incompatibility	Certain elemental compositions and the thermal cycle of the process may promote the formation of intermetallics and undesired hard phases, which at the same time may cause cracking of the built part.	Conflicting compositions should be avoided when there is a risk of formation of intermetallics. This can be prevented based on phase diagrams derived from CALPHAD (Calculation of Phase Diagrams) simulations.
Cracking–residual stresses	Differing thermomechanical properties (i.e., CTE, elastic modulus) or differences in the crystal structures of the constituents may cause additional residual stresses during processing or in-service operation. When high residual stresses are generated, the material will suffer a catastrophic failure as a result of cracking.	Preheating has been reported to reduce residual stresses. Conversely, FGM strategies can be implemented to mitigate the formation of residual stresses.

**Table 5:** Most relevant publications concerning the L-DED of MMC coatings.

Publications	Materials	Application	Main Results	Limitations/Observations
Jiang and Kovacevic, 2007 [139]	AISI H13 and TiC	Die and mould industry	MMCs containing less TiC exhibited higher erosion resistance.	No comprehensive discussion of the mechanisms behind this phenomenon was provided.
Nurminen, Näkki and Vuoristo, 2009 [138]	Various matrix and reinforcement materials	Miscellaneous	The abrasion resistance of the MMC does not depend solely on the reinforcement but also on the matrix.	The study focused only on the material selection and no importance was given to the processing conditions.
Bartkowski and Bartkowska, 2017 [125]	Stellite 6 and WC	Oil and gas	The massive difference in hardness between the reinforcement and matrix promoted severe wear mechanisms.	Preliminary results on the effects of different process parameters were provided, but no comprehensive analysis of the underlying phenomena was given
Muvvala, Patra Karmakar, and Nath, 2017 [126]	Inconel 718 and WC	Aerospace industry	The longer melt pool lifetime promotes the decomposition of the reinforcement phase and is detrimental to the wear resistance of MMC coatings.	The hardness of the coatings and subsequent hardening mechanisms are not evaluated.
Li et al., 2021 [122]	Fe60 self-fluxing alloy and WC	Miscellaneous	The phase evolution of the multi-material coating was formulated and supported by microstructural observations.	The effect of the processing conditions was not considered and the performance of the proposed coatings was not evaluated comparatively.
Zhao et al., 2022 [141]	Ni-based alloy and WC	Miscellaneous	WC particles suffer from dissolution, diffusion, fragmentation, and precipitation mechanisms when exposed to high temperatures.	The study only focused on microhardness and not on the hardness of the composite. The influence of the thermal cycle of the process was not considered.
Raahgini and Verdi, 2022 [120]	Inconel 625 and VC	Miscellaneous	Though showing higher hardness, MMC coatings with high reinforcement contents suffered a loss in wear resistance due to the appearance of the third body wear mechanism.	The effect of the processing conditions was not considered.

## Data Availability

Data sharing not applicable.
